# The associations between endothelial lipase 584C/T polymorphism and HDL-C level and coronary heart disease susceptibility: a meta-analysis

**DOI:** 10.1186/1476-511X-13-85

**Published:** 2014-05-22

**Authors:** Gaojun Cai, Zhiying Huang, Bifeng Zhang, Weijin Weng, Ganwei Shi

**Affiliations:** 1 Department of Cardiology, Wujin Hospital Affiliated to Jiangsu University, Changzhou, Jiangsu Province, China; 2 Department of Pediatrics, NO.2 Hospital of Changzhou, Changzhou, Jiangsu, China; 3 Department of Medicine, University of Chicago, Chicago, Illinois, USA

**Keywords:** Endothelial lipase, High density lipoprotein cholesterol, Polymorphism, Coronary heart disease, Meta-analysis

## Abstract

**Background:**

Studies had investigated the relationships between endothelial lipase (EL) 584C/T polymorphism and high density lipoprotein cholesterol (HDL-C) level and coronary heart disease (CHD), but the results were controversial. To investigate a more authentic associations between EL 584C/T polymorphism and HDL-C level, and the risk of CHD, we performed this meta-analysis.

**Methods:**

We searched electric databases for all articles on the associations between EL 584C/T polymorphism and HDL-C level, and CHD risk. Odds ratios (ORs) with 95% confidence interval (CI) were used to evaluate the strength of the association between the EL 584C/T polymorphism and the CHD susceptibility. The pooled standardized mean difference (SMD) with 95% CI was used for the meta-analysis of EL 584C/T polymorphism and HDL-C level. Begg’s funnel plots and Egger’s test were used to examine the publication bias.

**Results:**

For CHD association, the pooled OR was 0.829 (95% CI: 0.701-0.980, *P* = 0.028) for the dominant model and 0.882 (95% CI: 0.779-0.999, *P* = 0.049) for the allelic model. By meta-regression analysis, we found that only total sample size could influence the initial heterogeneity. When the subgroup analysis was carried out, we found that the protective effect only existed in the subgroups of relatively small sample size. Sensitivity analyses indicated that Tang’s study influenced the overall results significantly. We calculated the pooled ORs again after excluding Tang’s study and found the association between EL 584C/T polymorphism and the risk of CHD was not significant for any genetic model. For HDL-C level association, the carriers of 584 T allele had a higher HDL-C level than the non-carriers. The pooled SMD was 0.399 (95% CI: 0.094-0.704, *P* = 0.010). When the studies were stratified by ethnicity and total sample size, the positive effects existed in the Caucasians and in subgroups of larger sample size. No significant publication bias was found in the present meta-analysis.

**Conclusions:**

The results of the present meta-analysis suggest that the carriers of EL 584 T allele have a higher HDL-C level in Caucasian populations. Whereas, it might not be a protective factor for CHD.

## Introduction

Coronary heart disease (CHD) and its serious complications are among the most common causes of death in developed countries [1]. The pathogenesis of CHD is related to multiple risk factors, including environmental and hereditary factors. Recently, there has been an increasing interest in the role of the single-nucleotide polymorphisms (SNPs) in the pathogenesis of CHD. Some SNPs may be associated with the risk of CHD [2,3], and others may be not [4,5].

Endothelial lipase (EL), which was first discovered by two independent research groups in 1999, might increase the susceptibility to CHD [6,7]. EL protein is secreted mainly by vascular endothelial cells. It is a new member of the triglyceride (TG) lipase family, which has both phospholipase activity and TG lipase activity. A mature EL consists of three conserved catalytic regions and binding sites. A mature EL is about 55KDa. EL can hydrolyze the high density lipoprotein cholesterol (HDL-C) and then generate free fatty acids, lysolecithin and low-lipid ApoAI [8]. There is a growing body of evidence suggesting that EL plays a crucial role in the pathogenesis of CHD by reducing the HDL-C and inducing the macrophages to take up native low density lipoprotein cholesterol (LDL-C).

The coding gene for EL protein is located at 18q21.1. In 2002, the EL 584C/T gene variant (rs2000813) was first identified by deLemos et al., which leads to the amino acid substitution [9]. The thymine is substituted for cytosine at nucleotide position 584, leading to a change from Thr to Ile at the position 111 of the EL protein. In previous studies, the genetic variant frequency was reported differently in White and Black (31.2% and 10.3%, respectively), and varied significantly in different populations [10,11]. Several studies had investigated the relationships between EL 584C/T polymorphism and HDL-C level and/or the risk of and CHD [12-21]. But, the results were controversial. Some evidences indicated that this common variant might be associated with HDL-C level and also play an important role in the development of CHD [12,13]. In contrast, some other studies had contradictory conclusions [14-17]. In 2009, Jensen *et al*. reported that no significant association was found between this variant and the risk of CHD among Caucasian population in three independent populations [14]. In 2012, Cai *et al*. concluded that the EL 584C/T polymorphism was not associated with HDL-C level or the CHD risk in the Chinese population [15].

Because the sample size in each of the published studies was relatively small, we performed this meta-analysis to investigate whether there are real associations between EL 584C/T polymorphism and the HDL-C level, and the risk of CHD.

## Methods

### Studies selection

The meta-analysis followed the Perferred Reporting Items for Systematic Reviews and Meta-analysis (PRISMA) criteria [22]. We searched the PubMed, Foreign Medical Journal Service (FMJS), Google Scholar, Web of science, Embase, Wanfang Data ( http://www.wanfangdata.com.cn), and China National Knowledge Infrastructure (CNKI) databases for all articles on the associations between the EL 584C/T polymorphism with HDL-C level, and the CHD risk (last search was updated 1 February 2014). The following terms were used in the search: ("*endothelial lipase*" or "*EL*"), ("*polymorphism*" or "*mutation*" or "*variant*"), ("*blood lipid*" or "*HDL-C*") ("*coronary heart disease*" or "*coronary arterial disease*" or "*angina pectoris*" or "*myocardial infarction*" or "*acute coronary syndrome*" or "*CHD*" or "*CAD*" or "*AP*" or "*MI*" or "*ACS*"). The inclusion criteria were as follows: (1) The study examined the associations between the EL 584C/T polymorphism and HDL-C level and/or CHD risk; (2) For CHD association, the study must be case–control or nested case–control study and must have the clear original data of genotypic and allelic frequencies; (3) For HDL-C level association, the study must have clear original data of the mean of HDL-C level and standard deviations (SD) by genotypes. At the same time, the number of each genotype must be clear; (4) There was no restriction on language. References cited in the relevant papers were also scanned.

### Data extraction

Data from the eligible studies were collected independently by the two authors (Cai and Huang). Disagreement was solved with by a discussion between the two authors. The following data were collected from each study: first author’s name, year of publication, average age, country, ethnicity of the studied population, numbers of cases and controls, frequency of EL 584C/T gene polymorphism in cases and controls, the mean of HDL-C level and SD by genotypes. If a paper's data was unconvincing, we attempted to contact the correspondent author by e-mail. All the data were recorded in a standardized form.

### Data analysis

The odds ratios (ORs) with 95% CI were used to evaluate the strength of the association between the EL 584C/T polymorphism and the CHD susceptibility. The pooled ORs were performed for four genetic models (allelic model: T vs. C; additive model: TT vs. CC; recessive model: TT vs. CT + CC; and dominant model: TT + CT vs. CC). A fixed effect model (a Mantel-Haenszel method) was used to evaluate the results if the between-study heterogeneity was not significant (I^2^ ≤ 50%, *P* > 0.05), which was investigated and measured using Cochrane Q statistic. Otherwise, the random-effect model (a Dersimonian-Laird method) was used [23]. Sensitivity analysis was carried out by calculating the results again by omitting one single study each time. If there was significant heterogeneity among studies, we performed the meta-regression analysis to explore the sources of heterogeneity. The confounding factors included year of publication, ethnicity, RR (ratio of case size to control size), type of study and total sample size. Subgroup analysis was performed by ethnicity, total sample size and deviation from Hardy-Weinberg equilibrium (HWE). The pooled standardized mean difference (SMD) with 95% CI was used for the meta-analysis of EL 584C/T polymorphism and HDL-C level. The publication bias between the studies was examined by Begg’s funnel plots and Egger’s test (*P* < 0.05 was considered representative of statistically significant publication bias). HWE was assessed by Fisher’s exact test and a *P* value smaller than 0.05 was considered statistically significant. All statistical analyses were performed by using STATA version 12.0 (StataCorp LP, College Station, Texas 77845 USA).

## Results

### Studies characteristics

**Figure 1:**
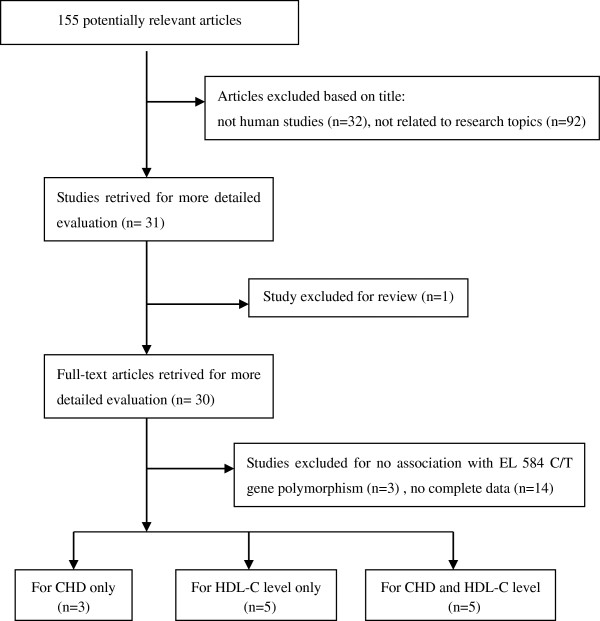
Flow diagram of article selection process for EL 584C/T polymorphism with HDL-C level and CHD risk.

There were 155 articles relevant to the search words (PubMed 46, FMJS 21, Wanfang 33, and CNKI 55), of which 142 articles were excluded. Of the 142 excluded studies, 124 articles were further excluded based on their titles (32 studies were not human studies and 92 studies were not related to research topics), one paper was a review [24] and three studies were not related with the EL 584C/T gene polymorphism [25-27] and 14 studies did not have complete data (Figure 1). A total of eight studies (nine cohorts) including 3036 cases and 4777 controls, which evaluated the relationship between EL 584C/T polymorphism and CHD, were involved in the meta-analysis. Main characteristics of these eligible studies were listed in Table 1. According to the data of all studies, the frequency of T allele was 29.4% among the cases and 33.7% among the controls. For the control subjects, the frequency of the T allele ranged from 11.7% to 50.0%. The total sample size in these case–control studies varies considerably (ranging from 214 to 1858). Among them, three studies came from Asia and the total sample size of each of these three studies was smaller than 600 [12,13,17]. The papers were published from 1992 to 2012. In the meta-analysis, four populations were Asians and the others were Caucasians. All the studies were case–control studies. But four studies were nested case–control studies and came from the Diet, Cancer, and Health (DCH) study, Nurses’ Health Study (NHS), Health Professionals Follow-up Study (HPFS) and EPIC-Norfolk study respectively [16,28-30]. Because the populations of DCH study were divided by gender, we treated men and women as two different cohorts. The diagnostic criteria of CHD were appropriated in all of these studies. The controls in three studies deviated from HWE [13,15,16]. In addition, a total of ten studies (11 cohorts) including 7602 individuals, which evaluated the relationship between the EL 584C/T polymorphism and the HDL-C level, were involved in the present meta-analysis. Table 2 lists the characteristics of these studies. Among them, eight cohorts were involved in Asian subjects and three cohorts were involved in Caucasians. Five studies were case–control studies and five studies were cohort studies.

**Table 1:** Main characteristics of studies involved in this meta-analysis of EL 584C/T polymorphism and CHD risk

**First author**	**Year**	**Ethnicity**	**Type of study**	**Mean age (years) (case/control)**	**Sample size (case/control)**	**Total sample size**	**Case (*N*)**			**Control (*N*)**			**MAF (control)**	**HWE (*P*)**
							**CC**	**CT**	**TT**	**CC**	**CT**	**TT**		
Rimm [30]	1992	Caucasian	nested C-C	66/66	262/519	781	129	117	16	240	239	40	0.31	0.06
Colditz [29]	1997	Caucasian	nested C-C	62/62	241/477	718	115	110	16	224	214	39	0.31	0.22
Shimizu [13]	2007	Asian	C-C	60/58	107/107	214	70	36	1	54	50	3	0.26	0.03
Zhu [17]	2007	Asian	C-C	65/60	242/196	438	186	56	0	150	46	0	0.12	0.06
Tjonneland^a^[28]	2007	Caucasian	nested C-C	60/56	235/763	998	116	102	17	391	312	60	0.28	0.84
Tjonneland^b^[28]	2007	Caucasian	nested C-C	58/56	763/880	1643	393	304	66	446	361	73	0.29	1.00
Tang [12]	2008	Asian	C-C	63/62	265/265	530	174	85	6	125	122	18	0.30	0.10
Vergeer [16]	2010	Caucasian	nested C-C	-/-	597/1271	1868	97	413	87	207	857	207	0.50	<0.01
Cai [15]	2012	Asian	C-C	63/60	324/299	623	195	116	13	168	122	9	0.23	0.02

^a^Women cohort in the study; ^b^Men cohort in the study; C-C: case–control; MAF: minor allelic frequency; HWE: Hardy-Weinberg equilibrium; *N*: number.

**Table 2:** Characteristics of individual studies included in the meta-analysis of EL 584C/T polymorphism and HDL-C level

**Author**	**Year**	**Country**	**Ethnicity**	**Type of study**	**MAF (%)**	**CC**			**CT + TT**		
						**n**	**Mean**	**SD**	**n**	**Mean**	**SD**
Cai [15]	2012	China	Asian	C-C	22.63	363	1.16	0.30	260	1.16	0.33
Zhu [17]	2007	China	Asian	C-C	11.64	336	1.15	0.35	102	1.28	0.36
Liu^a^[20]	2010	China	Asian	Cohort	27.36	325	1.58	0.40	320	1.67	0.45
Liu^b^[20]	2010	China	Asian	Cohort	32.05	264	1.85	0.48	374	1.89	0.50
Shimizu [13]	2007	Japan	Asian	C-C	21.96	124	1.22	0.04	90	1.23	0.04
Halverstadt [21]	2003	American	Caucasian	Cohort	25.90	44	1.17	0.05	39	1.22	0.05
Vergeer [16]	2010	European	Caucasian	nested C-C	35.58	1605	1.31	0.39	1573	1.36	0.39
Tang [12]	2008	China	Asian	C-C	45.19	299	1.01	0.04	231	1.12	0.06
Ma [33]	2003	American	Caucasian	Cohort	29.17	180	1.11	0.27	192	1.16	0.31
Hutter [19]	2006	Japan	Asian	Cohort	23.84	311	1.37	0.36	230	1.40	0.35
Yamakawa-Kobayashi [18]	2003	Japan	Asian	Cohort	24.12	198	1.40	0.27	142	1.40	0.28

MAF: minor allele frequency; HDL-C: HDL cholesterol; Liu ^a^Bai Ku Yao; Liu ^b^Chinese; C-C: case–control.

### Meta-analysis results

**Figure 2:**
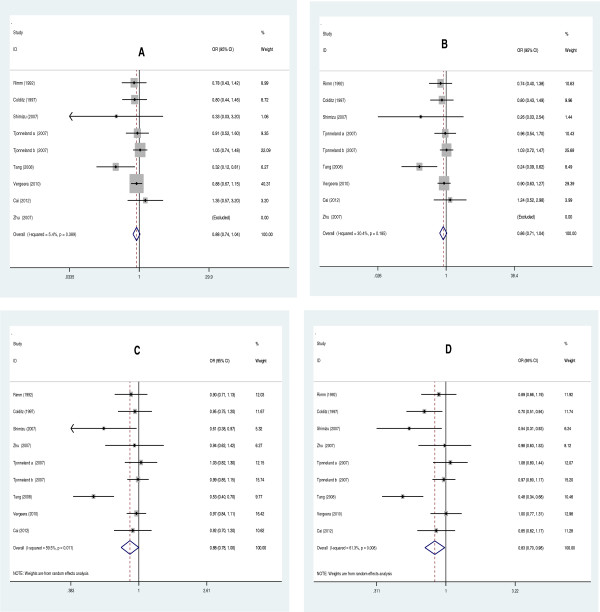
**Forest plots of CHD associated with distribution of genotypic frequencies of EL 584C/T. A**: recessive model; **B**: additive model; **C**: allelic model; **D**: dominant model.

**Figure 3:**
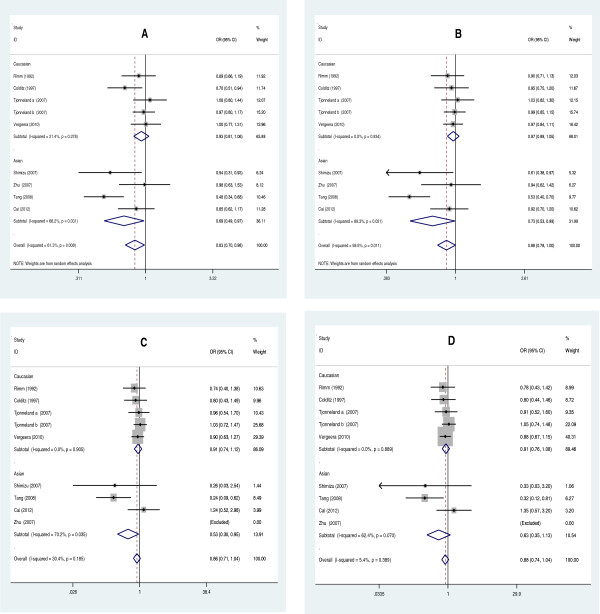
**Forest plots of CHD associated with distribution of genotypic frequencies of EL 584C/T stratified by ethnicity. A**: dominant model; **B**: allelic model; **C**: additive model; **D**: recessive model.

**Figure 4:**
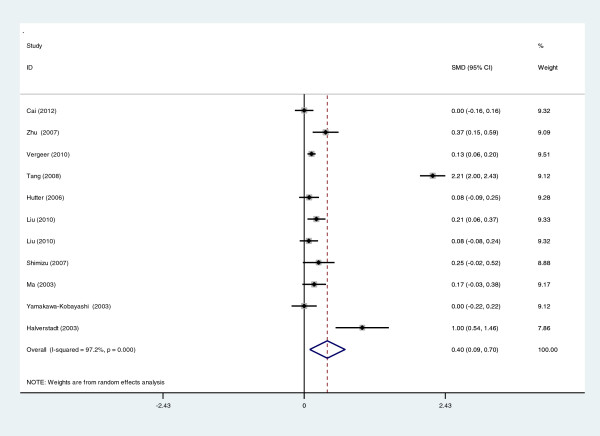
Forest plot of EL 584 C/T associated with HDL-C level in overall population (CT+TT vs. CC).

Table 3 lists the main results of the meta-analysis of the associations between EL 584C/T polymorphism and CHD risk. Overall, the pooled OR was 0.829 (95% CI: 0.701-0.980, *P* = 0.028) for dominant model and 0.882 (95% CI: 0.779-0.999, *P* = 0.049) for allelic model (Figure 2). When the studies were stratified by ethnicity, the positive results were found only in the Asian subgroups, but not in the Caucasian populations (Figure 3). The pooled OR was 0.83 (95% CI: 0.70-0.98, *P* = 0.034) in Asian subgroups for the dominant model, 0.727 (95% CI: 0.532-0.993, *P* = 0.045) for the allelic model and 0.529 (95% CI: 0.297-0.945, *P* = 0.032) for the additive model, respectively. For HDL-C level association, the carriers of 584 T allele had the higher HDL-C level than the non-carriers. The pooled SMD was 0.399 (95% CI: 0.094-0.704, *P* = 0.010) (Figure 4).

**Table 3:** Meta-analysis results of association between EL 584C/T polymorphism and CHD risk

	**Number of studies**	**Case sample**	**Control sample**	**TT vs CC + CT**				**CT + TT vs CC**				**T vs C**				**TT vs CC**			
				**OR(95%)**	***P***_**OR**_	***P***_**Q**_	***I***^***2***^**(%)**	**OR(95%)**	***P***_**OR**_	***P***_**Q**_	***I***^***2 ***^**(%)**	**OR(95%)**	***P***_**OR**_	***P***_**Q**_	***I***^***2 ***^**(%)**	**OR(95%)**	***P***_**OR**_	***P***_**Q**_	***I***^***2 ***^**(%)**
**Total**	9	3036	4777	0.876(0.738-1.041)	0.133	0.389	5.4	0.829(0.701-0.980)	0.028	0.008	61.3	0.882(0.779-0.999)	0.049	0.011	59.5	0.859(0.709-1.040)	0.120	0.185	30.4
**Ethnicity**																			
Asian	4	938	867	0.632(0.355-1.125)	0.119	0.070	62.4	0.687(0.486-0.971)	0.034	0.031	66.2	0.727(0.532-0.993)	0.045	0.021	69.3	0.529(0.297-0.945)	0.032	0.035	70.2
Caucasian	5	2098	3910	0.905(0.756-1.084)	0.279	0.889	0.0	0.928(0.813-1.060)	0.272	0.278	21.4	0.969(0.894-1.051)	0.454	0.934	0.0	0.912(0.745-1.117)	0.375	0.905	0.0
**Total sample size**																			
<600	3	614	568	0.319(0.134-0.761)	0.010	0.982	0.0	0.631(0.401-0.993)	0.047	0.042	68.4	0.659(0.463-0.937)	0.020	0.079	60.5	0.242(0.100-0.583)	0.002	0.955	0.0
≥600	6	2422	4209	0.920(0.772-1.098)	0.357	0.862	0.0	0.920(0.822-1.031)	0.152	0.371	7.1	0.965(0.893-1.043)	0.368	0.964	0.0	0.927(0.761-1.129)	0.452	0.914	0.0
**HWE**																			
Yes	5	2008	3100	0.859(0.681-1.085)	0.202	0.220	30.3	0.827(0.662-1.033)	0.093	0.005	69.9	0.879(0.736-1.049)	0.152	0.006	69.1	0.832(0.654-1.057)	0.132	0.083	51.5
No	4	1028	1677	0.898(0.695-1.160)	0.409	0.443	0.0	0.833(0.623-1.116)	0.221	0.132	50.7	0.891(0.733-1.083)	0.245	0.173	42.9	0.910(0.662-1.251)	0.563	0.434	0.0

### Evaluation of heterogeneity

For CHD association, there was a significant heterogeneity for the dominant model (*I*^2^ = 61.3%, *P*_heterogeneity_ = 0.008) and for the allelic model (*I*^2^ = 59.5%, *P*_heterogeneity_ = 0.011). To explore the sources of heterogeneity between the studies, we performed the meta-regression analysis by ethnicity (Asian or Caucasian), year of publication (before 2006 or after 2006), type of study (case–control study or nested case–control study), R/R (more than 1.0 or less than 1.0) and total sample size (more than 600 or less than 600). We found that only the total sample size could influence the initial heterogeneity (*P*_meta-regression_ = 0.008, for allelic model).

**Figure 5:**
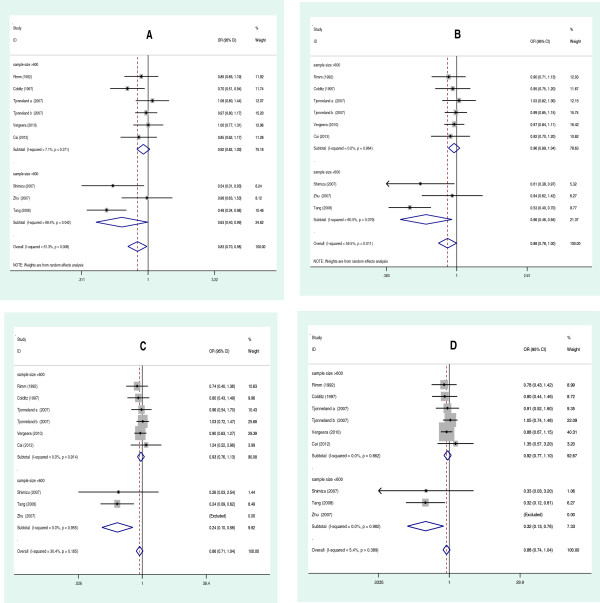
**Forest plots of CHD associated with distribution of genotypic frequencies of EL 584C/T stratified by sample size. A**: dominant model; **B**: allelic model; **C**: additive model; **D**: recessive model.

When the subgroup analysis was carried out by total sample size (more than 600 or less than 600), we found that the protective effect only existed in relatively small sample size subgroups. The pooled OR was 0.319 (95% CI: 0.134-0.761, *P* = 0.010) for the recessive model, 0.631 (95% CI: 0.401-0.993, *P* = 0.047) for the dominant model, 0.659 (95% CI: 0.463-0.937, *P* = 0.020) for the allelic model and 0.242 (95% CI: 0.100-0.583, *P* = 0.002) for the additive model, respectively (Figure 5). When the stratified analysis was performed by whether deviating from HWE, no significant association between the EL 584C/T polymorphism and the CHD in subgroups was found for four genetic models (*P >* 0.05).

For the HDL-C level association, the heterogeneity among studies was also significant (*I*^2^ = 97.2%, *P*_heterogeneity_ = 0.000). To explore the sources of heterogeneity, we performed subgroup analyses by ethnicity (Asian or Caucasian) and total sample size (less than 600 or more than 600), but the heterogeneity remained significant. The subgroup analyses suggested that the association between EL 584C/T polymorphism and HDL-C level only existed in Caucasian populations and in subgroups of large sample size (Additional file 1).

### Sensitivity analysis

**Figure 6:**
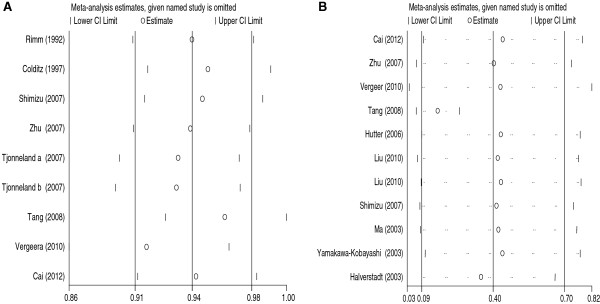
**Analysis of influence of individual study on the pooled estimate.** Open circle indicate the pooled OR or SMD. Horizontal lines represent the 95% CI. (**A**: For CHD association; **B**: For HDL-C level association).

The influence of a single study on the overall meta-analysis was carried out by calculating pooled ORs again by omitting one single study each time. Figure 6A showed the sensitivity analyses for CHD association for dominant model in the overall population. The results showed that the results changed greatly when Tang’s study was excluded. We calculated the pooled ORs again after excluding Tang’s study and found the association between EL 584C/T polymorphism and the risk of CHD was not significant for any genetic model (for the dominant model, OR = 0.908, 95% CI: 0.818-1.006, *P* = 0.066; for the recessive model, OR = 0.914, 95% CI: 0.766-1.089, *P* = 0.315; for the additive model, OR = 0.916, 95% CI: 0.753-1.115, *P* = 0.384; for the allelic model, OR = 0.952, 95% CI: 0.883-1.027, *P* = 0.203). Thus, the results indicated that Tang’s study influenced the overall results significantly.

For the HDL-C level association, the influence of each single study on the overall meta-analysis was also carried out by calculating pooled SMD again by omitting a single study each time. The results did not show any significant difference when omitting each study, which indicated that a single study didn’t influence the stability of the entire study (Figure 6B).

### Publication bias

**Figure 7:**
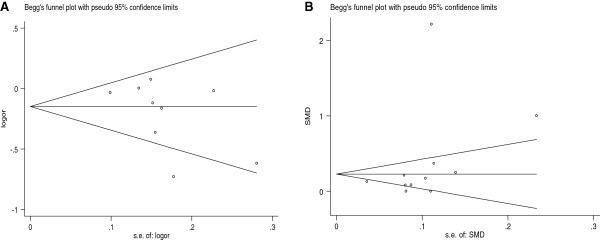
**Funnel plot of association between EL 584C/T with HDL-C level and CHD risk in overall study.** Each point represents a separate study for the indicated association. (**A**: For CHD association; **B**: For HDL-C level association).

The Begg’s funnel plot and Egger’s test were used to evaluate the publication bias of the literatures. Figure 7A displayed a funnel plot which examined the EL 584C/T polymorphism and overall CHD risk for the dominant model. No significant publication bias was found, which was confirmed by Egger’s test (*P* = 0.345 for the dominant model, 0.327 for the allelic model, 0.646 for the recessive model and 0.335 for additive model, respectively). For the HDL-C level, no significant publication bias was found, which was also confirmed by Egger’s test (*P* = 0.793) (Figure 7B).

## Discussion

In the present study, we performed a systematic review of the associations between EL 584C/T polymorphism with HDL-C level, and the risk of CHD. Our meta-analysis concluded that there was no significant association between the EL 584C/T polymorphism and the risk of CHD. Nevertheless, the carriers of EL 584 T allele had a higher HDL-C level than non-carriers in Caucasian populations.

A growing body of evidence indicates that the EL might play a crucial role in the HDL-C metabolism [31-33] and in the pathogenesis of cardiovascular disease (CVD) [34,35]. EL has a catalytic phospholipase activity and noncatalytic legend-bridging functions, which can hydrolyze the HDL-C and increase the clearance of HDL-C [36]. As we know, the level of HDL-C correlated with the risk of CHD negatively [37]. So the pro-atherosclerotic action of EL was probably partly caused by decreasing the level of HDL-C. The level of EL was regulated by several factors. Badellino et al. found the level of EL positively correlated with the level of high-sensitivity C-reactive protein, interleukin-6, soluble intercellular adhesion molecule-1, etc. but negatively correlated with the adiponectin level [38].

EL 584C/T gene variant is a missense polymorphism in exon 3, and was identified in 2002. To date, some studies have failed to validate the associations between EL 584C/T polymorphism and HDL-C level [13,15], and the risk of CHD [14-17], whereas other studies found this variant was associated with HDL-C level [12,17] and could also reduce the CHD susceptibility [12,13]. By the prospective case–control study in EPIC-Norfolk, Vergeer *et al*. suggested that the minor allele of EL 584C/T was not associated with CHD [16]. In our previous study, we didn’t find a statistically significant associations between the variant and HDL-C level, and the risk of CHD (OR = 0.92, 95% CI = 0.70-1.20, *P* = 0.528) either [15].

In 2008, Tang *et al*. carried out a study including 530 age- and sex-matched Chinese subjects to investigate the relationship between the common variant and the CHD risk [12]. They concluded that the T allele could significantly reduce the CHD susceptibility. At the same time, they found the serum HDL-C level was significantly higher in the T allele carriers (CT + TT genotypes) than the wide-type CC carriers. In a case–control study of 214 Japanese individuals, Shimizu et al. also found the T allele was an independent protective factor to AMI (OR = 0.52, 95% CI: 0.28-0.98, *P* = 0.04) [13].

In 2009, Jensen *et al*. performed a study to evaluate the relationship between the EL 584C/T polymorphism and the risk of CHD in three independent populations [14]. Their study did not support an association between this variant and the risk of CHD in Caucasian populations. But only three independent Caucasian populations with 4140 individuals were included in their study and all studies were nested case–control studies. The statistical effect was limited because of the relatively small sample size. So we performed this meta-analysis including 13 independent populations. The results of the present meta-analysis were more convincing, as the statistical power increases. In this study, we found the EL 584C/T polymorphism was not significantly associated with the risk of CHD. Although the pooled effects indicated that the EL 584C/T polymorphism might be significantly associated with CHD in overall population (for the dominant model, OR = 0.829, 95% CI: 0.701-0.980, *P* = 0.028; for the allelic model, OR = 0.882, 95% CI: 0.779-0.999, *P* = 0.049). The sensitivity analysis found that the pooled effects changed after Tang’s study was excluded, which indicated that this study influenced the stability of the whole study. When Tang’s study was excluded, the conclusion changed completely (for the dominant model, OR = 0.908, 95% CI: 0.818-1.006, *P* = 0.066; for the allelic model, OR = 0.952, 95% CI: 0.883-1.027, *P* = 0.203). In our study, we found the significant heterogeneity among studies (*I*^2^ = 61.3%, *P*_heterogeneity_ = 0.008, for dominant model; *I*^2^ = 59.5%, *P*_heterogeneity_ = 0.011, for allelic model). So, we performed the meta-regression analysis to explore the sources of heterogeneity. The confounding factors, involving ethnicity, year of publication, RR and total sample size, were involved in meta-regression analysis. Total sample size (more than 600 or less than 600), but not other factors, could influence the initial heterogeneity (*P*_meta-regression_ = 0.008, for allelic model; *P*_meta-regression_ = 0.027, for dominant model), which could explain most heterogeneity. When we performed the subgroup analysis by total sample size, we found the association only existed in relatively small sample size subgroups, rather than larger sample size subgroups. In addition, when the stratified analysis was carried out by ethnicity, we found the protective effect only existed in the Asian subgroups. But, the sample size of each Asian study ranged from 214 to 623, which was relatively small. Especially, the Tang’s study involved both Asian subgroup and small sample size subgroup. We analyzed their study and found the frequency of T allele was significantly higher in their study than in others and the controls were not all confirmed by coronary angiography. These might partly influence the heterogeneity and the results. We calculated the pooled ORs again after excluding their study. The pooled ORs suggested that the EL 584C/T polymorphism was not associated with CHD risk. So, we should interpret the results cautiously.

In addition, our study concluded that the carriers of T allele had the higher HDL-C level than the non-carriers. The subgroup analysis suggested the positive result only existed in Caucasian populations. Because of the significant heterogeneity among studies, the subgroup analyses were carried out by ethnicity and the total of sample size. It was regrettable that the stratified analyses did not reduce the heterogeneity significantly. Individuals included in this study had different genetic background and environmental factors. At the same time, the sample size of each study varied and the age difference among the studies was also relatively large. All of these might contribute to the heterogeneity. The subgroup analyses suggested that the association between EL 584C/T polymorphism and HDL-C level existed in Caucasian populations and in subgroup of large sample size.

There were several inherent limitations in this meta-analysis. Firstly, the sample sizes of some studies were relatively small and they might not have an adequate power to detect the possible risk for the EL 584C/T polymorphism. Secondly, this meta-analysis only involved the published studies. As we all know, the papers having negative result were probably more difficult to be accepted for publication. So the inevitable publication bias may exist in the results, although the Egger’s tests indicated no remarkable publication bias in our meta-analysis. Thirdly, the populations only come from Asians and Caucasians. Other ethnic populations should be involved in the future studies, such as Africans.

## Conclusions

Despite these limitations, the results of the present meta-analysis suggest that the carriers of T allele have the higher HDL-C level in Caucasians but not in Asians. Whereas, there is no significant association between the EL 584C/T polymorphism and the reduced risk of CHD. Due to the limitations of the current meta-analysis, studies in Asian and other populations with larger sample size should be carried out to confirm the results in the future.

## Competing interest

The authors declare that they have no competing interests.

## Authors’ contributions

GJC, ZYH, BFZ, WJW and GWS carried out the search studies and drafted the manuscript. GJC, ZYH and BFZ, participated in the design of the study and performed the statistical analysis. GJC, ZYH and BFZ conceived of the study, and participated in its design and coordination and helped to draft the manuscript. All authors read and approved the final manuscript.

## Supplementary Material

Additional file 1: Figure A Forest plots of EL 584C/T associated with HDL-C level stratified by ethnicity. (CT+TT vs. CC). **Figure B.** Forest plots of EL 584C/T associated with HDL-C level stratified by sample size. (CT+TT vs. CC). Click here for file
