# Supplementary material for: The associations between endothelial lipase 584C/T polymorphism and HDL-C level and coronary heart disease susceptibility: a meta-analysis
Source: Lipids Health Dis. 2014 May 22;13:85. doi: 10.1186/1476-511X-13-85 (PMC4041051; doi:10.1186/1476-511X-13-85)
Supplement: Additional file 1: Figure A — Forest plots of EL 584C/T associated with HDL-C level stratified by ethnicity. (CT+TT vs. CC). Figure B. Forest plots of EL 584C/T associated with HDL-C level stratified by sample size. (CT+TT vs. CC). [file 1476-511X-13-85-S1.doc]

**A**

**Supplement Fig A. Forest plots of EL 584 C/T associated with HDL-C level stratified by ethnicity. (CT+TT vs. CC)**

**B**

**Supplement Fig B. Forest plots of EL 584 C/T associated with HDL-C level stratified by sample size. (CT+TT vs. CC)**
